# A Model Minister

**Published:** 1856-05

**Authors:** 


### HALL'S JOURNAL OF HEALTH.

OUR LEGITIMATE SCOPE IS ALMOST BOUNDLESS: FOR WHATEVER BEGETS PLEASURABLE AND HARMLESS FEELINGS, PROMOTES HEALTH; AND WHATEVER INDUCES DISAGREEABLE SENSATIONS, ENGENDERS DISEASE.

VOL. III.] MAY, 1856 [NO. V

### A MODEL MINISTER

Was *John McFarland*, the pastor of a village church, in the wild woods of the West, a worthy pupil of Dr. Mason the elder, the giant theologian of his time. As is the master, so is the man. It was a favorite idea with him, as was the case with his great Teacher, and perhaps too, it was a main foundation of what he subsequently accomplished, that the Holy Bible was its own best expositor. He considered mere human reason as a false light on a dangerous coast, the quicksands of the soul; hence the study of the Bible was his highest gratification, and to get the young of his congregation to love its study, seemed to engage his constant thought. A weekly Bible class was as much a matter of course, as the Sunday morning sermon, while as regularly were the children of the congregation trained to the recitation of the Catechism. "The Bible and the Catechism," he used to impress upon us, "are the hope of Presbytery." If the children were not in their places, he made it his business during the week to ascertain the cause and to remove it. Well do we remember the regular family visitation of himself, an elder always along, for religious conversation and prayer, and inquiry as to doubts and fears, and coldness and backsliding, as to difficulties and discouragements. In all things except preaching, the elders were considered his alternates, in visiting the sick, in attending to the poor, in conducting religious meetings. As for having a male member of the church who could not lead in prayer, that was not to be thought of. He seemed to believe that a man who *could not* pray in public, *did not* pray anywhere else. At the same time, "short and to the point" was a favorite phrase.

"Short duties make devotion sweet,
And keep the attention up,"

was a piece of poetry which he taught us in our teens; we always supposed the rhyme was in the reason of the thing. It struck us then as being true poetry, for as a boy, we dearly loved short prayers, and short sermons too, (can't say that age has made any improvement, rather think the proclivities are intensified in the same direction.) He was famous for illustrating his positions. He frequently repeated that a church member remarkable for long prayers in public, was fire-proof against all hints and inuendoes respecting his infirmity. At last, making a visit to his minister, and purposing to remain all night, one of the most bitter of a cold winter, he was shown into a kind of closet for purposes of private devotion, and the key turned. In the course of a very few minutes, a decided knocking at the door was heard, but as decided an inattention was given, until the poor fellow concluded it was no use "knocking at *that* door any more," so he quietly resigned himself to fate and freezing. But precisely at the expiration of the time usually employed in his public prayers, the good minister appeared to release his prisoner, remarking that not wishing him to be disturbed by having less opportunity for private devotion than for public ones, which were really less important, he had locked the door, and hoped he had not come too soon.

Mr. McFarland was a man of sleepless energy. He not only had a regular weekly lecture, but he held social meetings in the houses of his parishioners at various places and distances within ten miles of town, and to secure a good attendance he used to say, if he took the pains to prepare himself and ride several miles to feed them with scripture food, it was as little as they could do, to attend to his ministrations. In addition to this, he taught that several successive absences from church services, unless in case of being from home or sickness, was a cause for church censure. And the more effectually to secure a regular and full attendance, he taught his people, that going to meeting was to be considered as a matter of course, and not only so, it was their duty to attend their own church; however great the distinction of men who might occasionally preach in other congregations in the village, it was their business not to go, if their own church was open, even if but for a prayer meeting. So that when it happened there could be no preaching in his own church on the Sabbath day, he required the Elders to conduct the services in the same manner as if he were present, only a sermon was read by one of them; in this way he contended that his people would be kept together, and not be straggling about as sheep without a shepherd.

He was remarkable for considering tne proprieties of things, irrespective of the tyrant, custom; so, in having a new house of worship built in 1822, he had the seats arranged in such a manner, that a person entering the church, should face the whole congregation. This made them come early, and relieved those who were in, from the necessity of turning round, as if the neck of the whole assembly were fixed on one hinge, whenever a person entered the door, to the great discomfort of the preacher. "Then," said he, "if a man leaves the house during service, he will have to face me, I can see who he is, and he'll not be likely to do it a second time, without a reason. But to break up the ugly habit of audible conversation before the commencement of services, he had it arranged that familiar hymns should be sung and an occasional prayer offered by some of the members; this also gave the diffident an opportunity of entering the great congregation without attracting special attention.

Another sentiment Mr. McFarland took great pains to inculcate was, the habit of giving for all charitable purposes, to give as a matter of course, to whatever object the eldership thought worthy of presentation. He believed that when a people became accustomed to giving, they would give less reluctantly and more liberally.

Mr. McFarland was a brave man; he was a second John Knox in that respect. Many a time was he threatened with personal violence, from his uncompromising hostility to the vices of the village and his denunciation of all wrong doing. It was a common thing for men to make the most solemn asseverations they would never go to hear him again, but they would soon forget it. The people knew him to be a hard student, that they were sure to feel instructed, and that his teachings were reliable; the result was, he had the largest congregations in the town; the piety, the intelligence, the respectability of the place were always his hearers. This excellent man was never personal in the pulpit; never played the buffoon there; no flight of fancy, no flippancy, no irreverence, were chargeable to his account: the feeling of his Ambassadorship was always present in the sacred desk. "If I came to you from any earthly court, I should feel my responsibility, and should claim your respectful attention to all I had to say, but I appear before you as an Ambassador from the King of Kings, and the weight of souls presses on me." Thus he felt the honor of his ministry; that no human position could raise him higher.

In private and social life, Mr. McFarland was always courteous in his bearing, instructive and entertaining in his conversation, having the rare power, to make you at once feel that you were at home with him.

When he felt himself in the line of duty, he feared nothing and nobody. When he believed himself standing on Bible ground, the uproar of a congregation, or town, or community, or state, caused him not to falter an instant. *Horace Holley*, the learned, the accomplished, the elegant, the admired of the *Elite* of Boston society, became the Reverend President of Transylvania University. His polished manner, his winning ways, his high erudition, won all hearts in the Athens of the West, as Lexington, Kentucky, was then called. He was the admired, the bosom friend, and frequent guest of HENRY CLAY, and there was high promise of a successful and splendid career before him. But this profound scholar, this accomplished man, although cradled in the land of the Pilgrim Fathers, was not a Puritan or a Presbyter; his published discourses savored of Heresy, as to the fundamental point of Orthodox Faith. Mr. McFarland saw it, and although President Holley, in his triumphal car, was running away with all hearts, the ladies adoring, and the gentlemen, it is said, had their crowns shaven, as Mr. Holley was a little bald,—under such circumstances, our Model Pastor, the minister of a hundred members, in an obscure little inland town of a thousand inhabitants, put on his armor, and like the shepherd boy of olden time, threw down the challenge against the Goliath of his day. Sancho Panza, battling with a gate-post, or a fice dog barking at the moon, could not have inspired feelings of more sovereign contempt, than were excited in the mind of Mr. Holley and his admirers, towards their almost unknown opponent. But with his Bible and the right, with his logical criticisms, his sworn facts, his incontrovertible histories, his verbatim quotations, his irrefutable and clear-drawn conclusions, thrown out before the people in newspaper articles, in printed sermons and in a serial published by himself called *The Literary Pamphleteer*, the all-conquering President was driven back towards his Boston home, a dying, because, as was said, he was a disappointed man; that home, he never lived to see.

But there was another brave thing that Mr. McFarland did, a bravery which few ministers dare, perhaps not one in a thousand; it was in reference to his salary. He felt his power, and was not afraid to use it. His mode of presenting the subject was in substance as follows:

You have promised me a certain annual amount for preaching to you. You are Christian men, and should be men of veracity and honor. Your word should be as good as your bond. It is a debt which you have agreed to pay. That debt should be paid on or before the expiration of the current year. It should be *all* paid. A withholding of the last cent is as certainly a violation of a principle of right as the withholding of a larger part, or even the whole. None of you would think of going into bank and taking up a note the day after it was due, nor of getting that note by paying all except a few dollars or cents. The church has higher claims upon you, as Christian men, than any human corporation, for it is a divine institution. It has been said that I have money, and can do without a salary. That is true. But if a man, richer than you are, works for you, or sells you an article of value, you do not expect to have his labor or property for nothing, on the ground that he does not need it. Therefore, if I preach for you, I must be paid, on or before the expiration of each year, the last farthing of my salary. I hold the Church Session bound for it. They have gone your security, otherwise I should not have come among you. I know what congregational promises to pay are, so it is immaterial to me whether you pay or not; if you do not, the Session will. But if you do not refund to them, I will leave you, and the following are reasons which I hold all-sufficient: The Bible declares that *the laborer is worthy of his hire.* I will not countenance the violation of a positive Bible precept. If you are remiss in paying me, I will not suffer, because I have means of my own. But if you do not pay me for my labor, you will fall into a bad habit, and soon begin to think that a preacher ought not to be paid at all; and when I die, as there are few ministers who are not poor, very poor, my successor, however poor, will be treated as I would have been had I allowed myself to work for you for nothing and find myself into the bargain; so for the sake of those who come after me, for my own sake, and for the sake of a Bible principle, I shall expect my salary to be paid to the last cent, and within the year for which it is due. More than a third of a century ago, while we had not yet entered our teens, were these teachings delivered, but in spirit and idea we remember them well, and to this hour, no minister has ever labored in that congregation who has not been paid to the uttermost every farthing of his salary. Make a note of this, ye modern men of Galilee, ye ministers of the Word, and stand square up on the Bible Platform, and fear not that the preaching of the truth will fail to *accomplish that whereunto it is sent*, EVEN IN *the midst of a wicked and perverse generation.* If the people do not pay you promptly and fully, appeal bravely *to the law and to the testimony*, and fear no evil, for God will be with you.

Towards the last of his life, Mr. McFarland alienated the affections of some of his warmest and best members, and his death did not wholly heal the breach. The majority of the whole State Synod voted him hobby-horsical.

He thought that all baptized children were members of the church, and that they were neglected as to their religious training. He at once instituted a parochial school, perhaps the first in the United States, where the children of the church could be prepared for college under Presbyterian teachings wholly. Other children may come in—they are welcome—but our children must. Prayer and the reading of the Bible were daily duties in the school, and once a week, a Bible class. This was the first step. In the estimation of some, it savored of sectarianism and exclusiveness, and *many were offended thereat and walked no more with him.* He seemed to stand almost alone, and we have seen him weep in the presence of a whole State Delegation, at his isolated position; he saw he was ahead of his day and generation, but instead of seceding, he took another step forward, that it was the duty of al. baptized children to come to the communion as soon as they arrived at the years of discretion, which he never fixed, leaving that to be decided by the lights afforded at the time of action in each particular case. If they did not come to the communion, he considered it the duty of the eldership, the parents and the minister, to talk with them, to instruct them, to encourage them, and to warn and admonish them *with all long-suffering and patience*, until they were brought to a sense of duty. He did not hold that in any given case, even for outrageous conduct, they should be excommunicated, but that ceaseless and persevering effort should be made to induce the children of the church to live up to its privileges and its requirements, as Christian birth and baptism gave them a full title to all the church ordinances, and that no human power could debar them from the same, without exceeding their authority. But the mass of the people, with the eldership and clergy, either could not, or would not, understand him, or felt that it was not expedient to press these views at that time. The young men of the congregation were encouraged to conduct prayer-meetings and occasionally to give short addresses themselves, especially when he was not present; in this way a habit and facility of public speaking were imperceptibly formed, a need of previous thought and preparation gradually impressed itself on their minds, with the attendant consciousness of increased responsibility, and before they were aware of it, they were half clergymen, and thus it was that a large number of them eventually entered the ministry.

We will now state some of the practical results of such a pastorate.

1. More than one-half of all the male members of his Bible classes, of our time, became clergymen and missionaries.

2. Nine-tenths of those members now living are church members.

3. At one Synodical Session, if we did not hear amiss, more money was raised for benevolent purposes in a single year in Mr. McFarland's congregation, than in all the other congregations of his sect in the State.

4. That congregation, we believe, remains to this day, according to its means and numbers, the most liberal in the State.

5. The ministers to that people have been fully paid to this hour.

6. By multitudes of those who heard him oftenest, and saw most of him, his memory is cherished with a most profound respect, bordering on reverence, and with an affection like that which David bore towards Jonathan.

7. "*As Mr. McFarland used to say*," is a household phrase to this day among the families of his charge which yet remain.

8. More young men, it is believed, were sent into the ministry from his congregation, during his pastorate, and resulting from it, than from any dozen other congregations during the same time in the whole State. We appeal to every clerical reader, to every warm-hearted Christian, is not such a result worth living for?

Mr. Carper desires us to inform him, if agreeable, and wholly convenient, what all this has to do with a *Journal of Health;* that he became a subscriber with the view of deriving instruction as to some of the best means of preserving his health, and that the space occupied about a "Model Minister" is so much of his property appropriated to other purposes without his consent, and is therefore clearly a wrong.

That may be true, or not, Mr. Carper, but you have forgotten an item or two. Please remember that this Journal was undertaken in the first place to please ourselves; in the second place, to profit ourselves, and in the third and last place, to benefit you and others like you, as far as may be done by what pleases and profits us.

By the way, please try on these leathern spectacles, part leather, some part caoutchouc, rather given to stretching. Looking in another direction, you will perceive that Mr. McFarland has been in his grave a third of a century; he died in his prime. In a few years he did more than forty ordinary ministers do in a lifetime; he was one of the most valuable "*hands*" in the "*field;*" he ought to have been alive, and hearty and well, an active laborer in his Master's vineyard to this hour, but he is not, consequently the church is a loser of his services for thirty-three years. Look a little further and you will perceive that his death was the result of *dyspepsia;* that dyspepsia is always founded on injudicious eating, upon which point this Journal has not ceased to give *line upon line and precept upon precept,* from the first hour of its issue. Therefore, we believe that the obituary we have given of the pastor of our childhood affords a lesson to the clergy of this country, more impressive than a thousand pages of precepts about health, helping us to engrave it on the memory of every reader, as if carved in lines of brass, that *cutting short our usefulness by drinking brandy is not more a crime than accomplishing the same object by eating contrary to the lights of our time.*

It is hard eating, and not hard study, which lays multitudes of our clergy on the shelf long before *the burden and the heat of the day* come on. Fast eating, injudicious eating, unconscious eating, by which we mean bolting down our food when the mind is away from the body, on 'change, in the counting-room, on the stock list, on the sermon in preparation or just delivered, this is a bane of the age among the active and working men of the time, and whether a man has died of brandy or of beef steak administered to himself *ad libitum*, the suicide is not less a fact. Ministers tell us that if any man dies in the persistent practice of a known wrong doing, *there is no hope in his death;* and not less equivocal are they in their denunciations of those who have the light, but see it not, taking for their guide the Master's utterance, *seeing they see not, and hearing they hear not.*

For our own part, we can perceive no sufficient reasons why divine principles applied to morals should not hold good and be as safe as applied to our physical constitution. That is, we believe that a man who eats himself to death, against the experience and lights of the times in which he lives, whether he sees those lights and fails to use them, or whether having those lights he will not open his eyes to make use of them, the crime is just the same, the punishment under a righteous administration just as inevitable, and the minister or man who reads this article and gives it the "*go by*" will sooner or later be held to a strict accountability for the same, to the Judge of the whole earth. We tell you, reader, of two men on a magazine of war material, with the train thereto already fired, he who is asleep, unconscious of his danger, and he who is wide awake, but ignorant of that danger—their equal death is certain; in ignorance or unconsciousness, there is no salvation. As illustrative and instructive, we here give one letter from many of a similar character received by us in our professional, not editorial capacity, from

### A SUFFERING CLERGYMAN.

"For the last twenty-seven years, I have been the pastor of this church; and have been afflicted with dyspepsia and constipation for many years. For the last three years I have been compelled to live daily on bread made of unbolted flour, a small portion of lean meat, and a glass of buttermilk. I am compelled to take *Pepsin* or pills almost every day, or suffer, waking up at midnight with restlessness, heat in hands and feet, lasting for an hour, and with various other ill feelings finally fall to sleep again. Night after night I have to go through the same process. I have taken a great variety of pills, bitters and other medicines, giving temporary relief, the symptoms returning as soon as I leave off taking medicine. After the burning of the hands and feet continue about an hour, there is a sensation of trickling down within me, along the bowels like a little loose dirt on the side of a hill; this continues to increase, making me exceeding restless, and I turn from side to side, without relief, until all the food has passed down, and I fall asleep."

Let the reader reflect a moment on the wretchedness of that man's life, physic or purgatory, every day of his existence. It is useful to inquire into the antecedents of this unfortunate condition of things. "Indigestion and constipation for many years." These things are always brought on by unwise modes of life, and in no other way. In the case before us, there had been a persistent violation of the plainest and most fundamental laws of our physical being for nearly a quarter of a century. Does human law excuse a man from its infraction, on account of ignorance? It is held an aggravation in every intelligent community; neither will ignorance of the laws of our physical constitution make a way of escape from violation. If we are sick, it is our own fault; neglect and ignorance and appetite and passion have made us so; and yet, when ministers die in the midst of their usefulness, it is published as *a mysterious and inscrutable dispensation of Divine Providence.* What profanation! Might just as well poke your finger in the fire, then charge the suffering to the Almighty. Away with such logic, from this time forth and forever more, and be assured that if ever you get sick from this day, the reason of it will be, three times out of four, that you have made a beast or fool of yourself.

